# Recent Advances in Electrically Stimulated Insulin Delivery Systems

**DOI:** 10.1021/acsomega.5c06147

**Published:** 2025-09-02

**Authors:** Carlos Alemán, Helena Muñoz-Galán, Maria M. Pérez-Madrigal

**Affiliations:** † Departament d’Enginyeria Química, Campus Diagonal Besòs (EEBE), Universitat Politècnica de Catalunya, Av. Eduard Maristany 10-14, 08019 Barcelona, Spain; ‡ Barcelona Research Center for Multiscale Science and Engineering, Campus Diagonal Besòs (EEBE), Universitat Politècnica de Catalunya, Av. Eduard Maristany 10-14, 08019 Barcelona, Spain; § Institute for Bioengineering of Catalonia (IBEC), The Barcelona Institute of Science and Technology, Baldiri Reixac 10-12, 08028 Barcelona, Spain

## Abstract

Diabetes mellitus has become one of the greatest medical challenges affecting millions of people globally. Non- and minimally invasive approaches for insulin release are currently being intensively investigated to improve the treatment efficacy and quality of life for diabetic patients. Electrically triggered drug release exhibits tremendous potential since it allows medications to be dosed intermittently on demand and over a long period of time using simple, safe, and inexpensive approaches. Despite such advantages, the use of electrical signals has been mainly focused on the delivery of small drugs, with the administration of protein-based drugs, such as insulin, being addressed only sporadically. However, in recent years, the controlled release of insulin through electrical stimulation has begun to be seriously studied, attracting interest because of its capacity to reduce the incidence of hyperglycemia, which further reduces the potential complications in diabetic patients. This review examines the state of the art of electroregulated insulin delivery systems, discussing the current different approaches existing and analyzing the advantages and disadvantages of each one of them.

## Graphical abstract

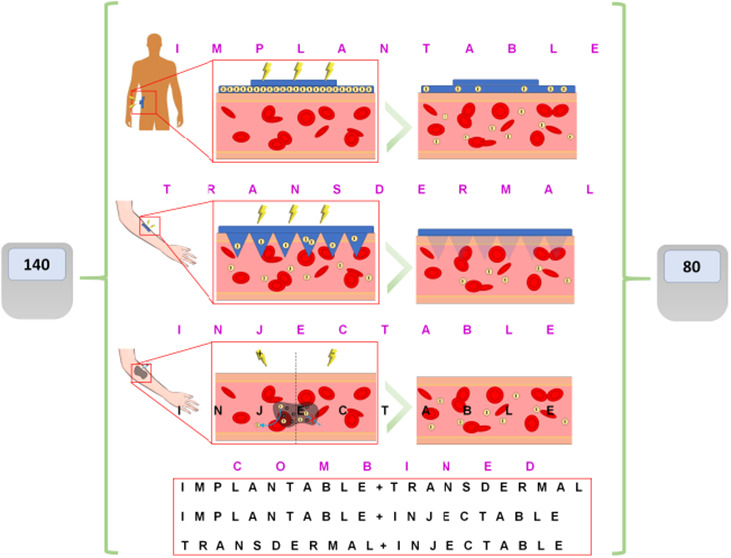

## Introduction

Diabetes mellitus is a metabolic disorder characterized by increased blood glucose levels, known as hyperglycemia. This can be attributed to a malfunction in the body’s production or use of insulin, a small protein hormone. Insulin, which is released by the pancreas, plays a crucial role in regulating glucose metabolism. Type 1 diabetes is characterized by severe insulin deficiency resulting from chronic and progressive destruction of pancreatic β-cells by the immune system, while in type 2 diabetes, the pancreas makes less insulin progressively with time and the body becomes resistant to insulin.

According to data provided by the International Diabetes Federation, the global incidence of diabetes reached 537 million people in 2021, and projections point to a further increase to 783 million people by 2045.^1^ Approximately, one in ten adults around the world is currently living with diabetes, and this figure is expected to go up to one in eight adults in 2045.^2,3^ Although type 2 diabetes accounts for about 90% to 95% of all diagnosed cases of diabetes,^4^ both diabetes types represent a significant challenge to public health on a global scale, leading to substantial mortality and healthcare expenses.^5^ Indeed, diabetes ranked eighth among the top 10 causes of death in 2021 (Figure 1).^6^ In addition, people with diabetes are at higher risk of developing other chronic diseases, such as cardiovascular disease, kidney disease, and blindness.^7–9^ Addressing diabetes prevention and control is, therefore, crucial to improving the health and well-being of individuals and communities around the world.

**Figure 1:**
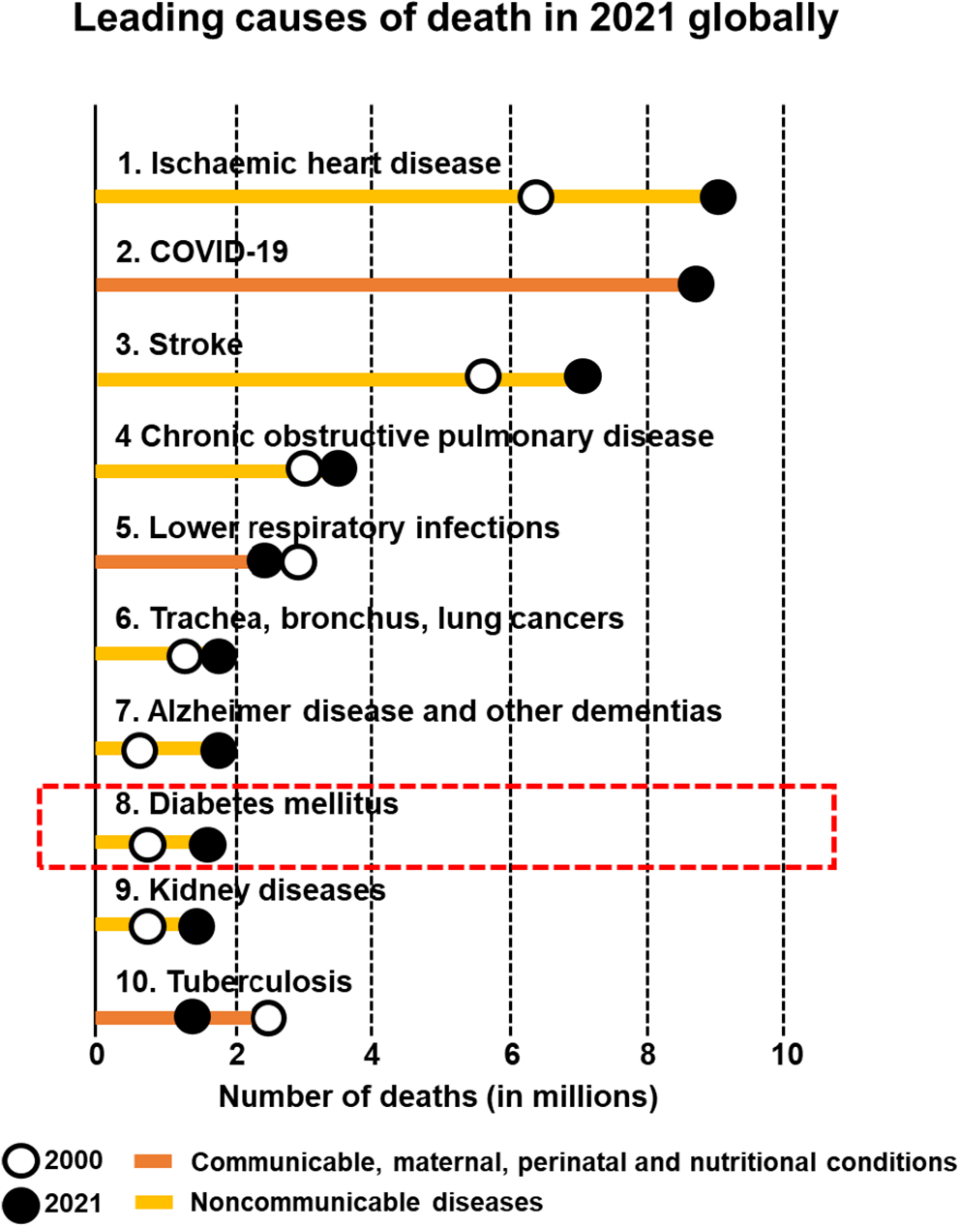
Top 10 leading causes of death globally. Adapted from ref. 6

Glucose monitoring is essential for the proper management of diabetes.^10,11^ Although traditional monitoring methods require multiple fingerstick measurements, which are invasive and uncomfortable, recent advances in glucose monitoring have addressed this issue, and several noninvasive options are already in clinical trials or available on the market. For example, noninvasive, wearable skin patches for continuous glucose monitoring, which operate through the utilization of mild electrical current to facilitate the transdermal extraction of glucose molecules, have been reported.^12,13^ Another product is GlucoTrack, which monitors glucose levels via the earlobe,^14,15^ by integrating ultrasonic, electromagnetic, and thermal technologies. More specifically, ultrasonic waves are utilized to measure variations in earlobe thickness, which correlate with glucose levels, while electromagnetic and thermal assessments are conducted to evaluate the dielectric properties and temperature of the earlobe, respectively.^14,15^ An alternative is the electrochemical detection of glucose in biological fluids, for example, sweat.^16,17^

In addition to improving patient comfort and compliance, innovations in glucose monitoring also enable effective management of diabetes through real-time continuous and noninvasive scanning. Recent review papers highlight the progress in noninvasive glucose-sensing technologies and their impact on diabetes care,^13,18,19^ while Table 1, which lists some noninvasive glucose monitoring technologies currently available on the market, illustrates how such advances have become a practical and accessible option for the population affected by diabetes.

**Table 1:** Nonexhaustive Overview Summarizing Representative Noninvasive Glucose Monitoring Technologies Currently Available in the Market

device	marketer	characteristics
SugarBeat	Nemaura Medical	thick disposable patch (1 mm) painlessly attached to the user’s arm, leg, or abdomen. Connected to a small electronic sensor, the patch measures the body’s interstitial fluid extracted from the skin every 5 min.^a^
Glucotrack	Glucotrack	earlobe clip and receptor with a large screen, where the history of readings is stored and can be seen. The ear clip takes 1 min to provide the result and must be replaced every 6 months.^b^
Dexcom	Novalab	a one-touch applicator easily inserts a small sensor just beneath the skin to continuously monitor glucose levels. Data are wirelessly sent to a display device through a transmitter.^c^
Medtronic Minimed	Medtronic Iberica	closed-loop glucose monitoring system that automatically adjusts insulin delivery to correct glucose levels every 5 min, 24 h a day, 7 days a week.^d^
FreeStyle Libre2	Abbot	applied to the back of the arm, continuously measures (every min) the concentration of glucose in the body’s interstitial fluid. It is water resistant.^e^
Eversense CMG System	Ascensia	sensor implanted in the arm that measures glucose using fluorescence-labeling technology.^f^

a https://www.medicalexpo.es/prod/nemaura-medical/product-112238-739565.html

b https://glucotrack.com/cbgm-technology/

c https://www.novalab.es/

d https://hcp.medtronic-diabetes.com.au/

e https://www.freestyle.abbott/bh-en/

f https://www.ascensiadiabetes.com

Diabetes treatment typically involves a combination of lifestyle modifications such as regular exercise, a healthy diet, and medication. Insulin administration is the main treatment for patients with type 1 diabetes,^20–22^ while some alternatives have been proposed for type 2 diabetes.^23–28^ For example, glucagon-like peptide 1 (GLP-1)-based therapies are used to treat type 2 diabetes since, under hyperglycemic conditions, GLP-1 stimulates insulin secretion and normalizes blood glucose levels, while it does not stimulate insulin secretion at normal glucose levels.^23^ Furthermore, GLP-1-based therapies also exhibit cardiovascular safety, which is particularly relevant given the intersection between diabetes and cardiovascular disease.^24^ Other effective therapies are based on the use of metformin,^25^ tirzepatide,^26^ thiazolidinediones,^27^ sulfonylureas,^28^ among others. Nevertheless, since noninsulin therapies tend to fail over time because of the progressive decline of insulin secretion,^29^ insulin administration becomes a necessity also for type 2 diabetes patients.

The most common method of insulin administration is subcutaneous injections, which deliver insulin subcutaneously using vials and syringes, insulin pens, and insulin pumps.^30,31^ The major drawback of such insulin administration methods is their invasive nature, which is typically associated with injection pain, needle phobia, lipodystrophy, noncompliance, and peripheral hyperinsulinemia. Comparative analysis of insulin injections and pump therapies has highlighted the advantages and disadvantages of both therapeutic devices.^32–35^ Although the use of insulin pumps is related to lower glycosylated hemoglobin (HbA1c) levels and fewer hypoglycemic events compared to multiple daily injections,^32^ their use remains hampered by their high cost, relative bulkiness, glucose detection lag time, need for recalibration, and risks of infection and ketoacidosis.^33^ Additionally, air bubbles in the pump tubing system or a blocked cannula can cause insufficient insulin delivery, potentially causing dangerous hyperglycemic episodes if the effective dose is slightly altered.^34,35^ Despite such limitations, the insulin digital therapy device market was anticipated to generate $2,082.3 million in revenue for 2025.^36^

The development of new insulin delivery technologies to control blood glucose levels while simultaneously improving patient compliance is a matter of social and economic interest that has received significant attention in recent years.^37–40^ Although the use of smart materials for insulin delivery using glucose-responsive mechanisms is being explored in this context, they often result in rapid and insufficient insulin release.^41–43^ Once again, these limitations highlight the need for advanced approaches capable of offering precise and controlled insulin delivery with minimal invasion.

Among smart materials, stimuli-responsive polymers have been extensively researched to develop controlled drug delivery systems (DDSs).^44–54^ The most popular stimuli-responsive polymers are those sensitive to light, pH, temperature, or voltage, even though electrically responsive systems stand out due to their precise control over drug release, well-developed instrumentation, and potential for miniaturization into implantable devices.^51–53^ These materials are stimulated by the application of an electrical field (either as current or voltage in low dosage) to enhance their inherent responsive attributes. Furthermore, the electric field for controlled drug release can be easily generated through external electro-conducting skin patches, miniaturized implants, and even with wireless strategies.^54–57^ Among the different electroresponsive materials used to release drugs, neat conducting polymers,^51,53,54^ electroactive hydrogels,^52,56,58^ and ionized materials^59–62^ are particularly promising because their response to the applied electrical field has great implications on the mechanism and tunability of release kinetics.

This Perspective discusses recent therapeutic advances and cutting-edge innovations in electrically stimulated insulin delivery systems. The electrostimulation approaches described herein have been demonstrated to effectively modulate the pharmacokinetics of insulin administration, offering more precise control over dosage and timing. By harnessing the unique capabilities of bioelectronics, electrical stimulation-based approaches provide a multifaceted strategy to address the complex challenges associated with diabetes management, which results not only in improved insulin absorption and glucose regulation but also in significant improvements in overall patient outcomes, thus representing a promising direction for the future of diabetes care.

The discussion of recent advances has been organized according to the delivery strategy. The next section is devoted to implantable devices designed for sustained and long-term insulin delivery. After this, transdermal DDSs, which provide a less invasive method of delivering insulin through the skin barrier, are discussed. This technology offers a convenient alternative to traditional injections, and some interesting approaches to electroregulate insulin release have been developed recently. The following section is dedicated to electroactive injectable insulin carriers such as hydrogels and nanoparticles (NPs). These systems represent a minimally invasive solution for the sustained and timed delivery of insulin through the application of electrical stimulation, providing prolonged therapeutic effects with minimal patient intervention. Finally, the most complex advances that simultaneously combine several of the aforementioned approaches are briefly presented and discussed. At the end of the review, we present the conclusions that can be drawn from this analysis and discuss future outcomes that require further attention from both the researchers’ and patients’ perspectives.

## Implantable Devices

Implantable electroactive systems have emerged as promising platforms for achieving controlled, on-demand insulin release. Among these, multilayered electrodes have demonstrated notable success due to their ability to integrate functional materials and respond to external stimuli, particularly electrical signals. These devices are typically constructed with different materials by using layer-by-layer-based technologies. By incorporating electroactive materials with redox properties into one or more of these layers, the electrostatically held insulin can be released by simply altering the strength of such interactions through electrical stimulation (*i.e*. changing the redox state of the electroactive material). It is worth mentioning that this strategy takes advantage of insulin’s net charge. The isoelectric point (IP) of insulin varies from 5.3 to 6.4, depending on the source, and, therefore, insulin is mainly negatively charged at physiological pH, while it is positively charged when the pH is lower than the IP.^63^

In an early study, Shameeli and Alizadeh^64^ engineered an implantable insulin delivery system based on films of electropolymerized polypyrrole (PPy) nanowires and immobilized gold NPs, which were functionalized with thioglycolic acid for the insulin loading. Thus, such a delivery system was responsive to both electrical voltages and pH variations. The release of insulin at a neutral pH was promoted by the electrical stimulus. While the application of negative potentials enhanced the release, positive potentials slowed it down. The protonation of thioglycolic acid at low pHs also reduced the release rate because of the formation of strong hydrogen bonding interactions with insulin, as well as the interaction with PPy chains when a negative electrical potential was applied.^64^ Overall, such a dual-responsive platform, which was also proposed for oral administration, was designed to reduce the delivery of insulin in the stomach (*i.e*. the pH of acid gastric juice is 1.2) and to enhance it in neutral environments like the intestines (*i.e*. the pH of intestinal fluids is 6.8) using electrical stimuli.

Szunerits and co-workers^65^ developed a system consisting of a positively charged composite layer, which was made of an insulin-impregnated matrix of reduced graphene oxide (rGO) loaded with nickel hydroxide nanostructures (rGO/insulin/Ni­(OH)_2_). The rGO/insulin/Ni­(OH)_2_ layer was prepared onto a gold-coated glass substrate (rGO/insulin/Ni­(OH)_2_/Au) using a single-step process. This consisted of a previously developed electrophoretic deposition approach that is sketched in Figure 2a (*i.e*. a DC voltage of 1.5 V was applied for 5 min to a solution of graphene oxide, insulin, and NiCl_2_·in water/ethanol (1:1)).^66^ At the physiological pH, the amount of insulin loaded into the electrophoretically deposited layer was 48 μg/mL. The release of loaded insulin was achieved by applying a negative potential to the rGO/insulin/Ni­(OH)_2_/Au electrode, which was optimized in previous work at a value of −0.8 V (Figure 2b).^67^ Such voltage, which resulted in an insulin release of 70% ± 4% in a physiological medium, charged the interface negatively and induced a burst insulin release due to the repulsive electrostatic interactions between the interface and the loaded drug molecules. Conversely, when a positive voltage was applied, the release of insulin was very low, and no burst release was observed (Figure 2b).

**Figure 2:**
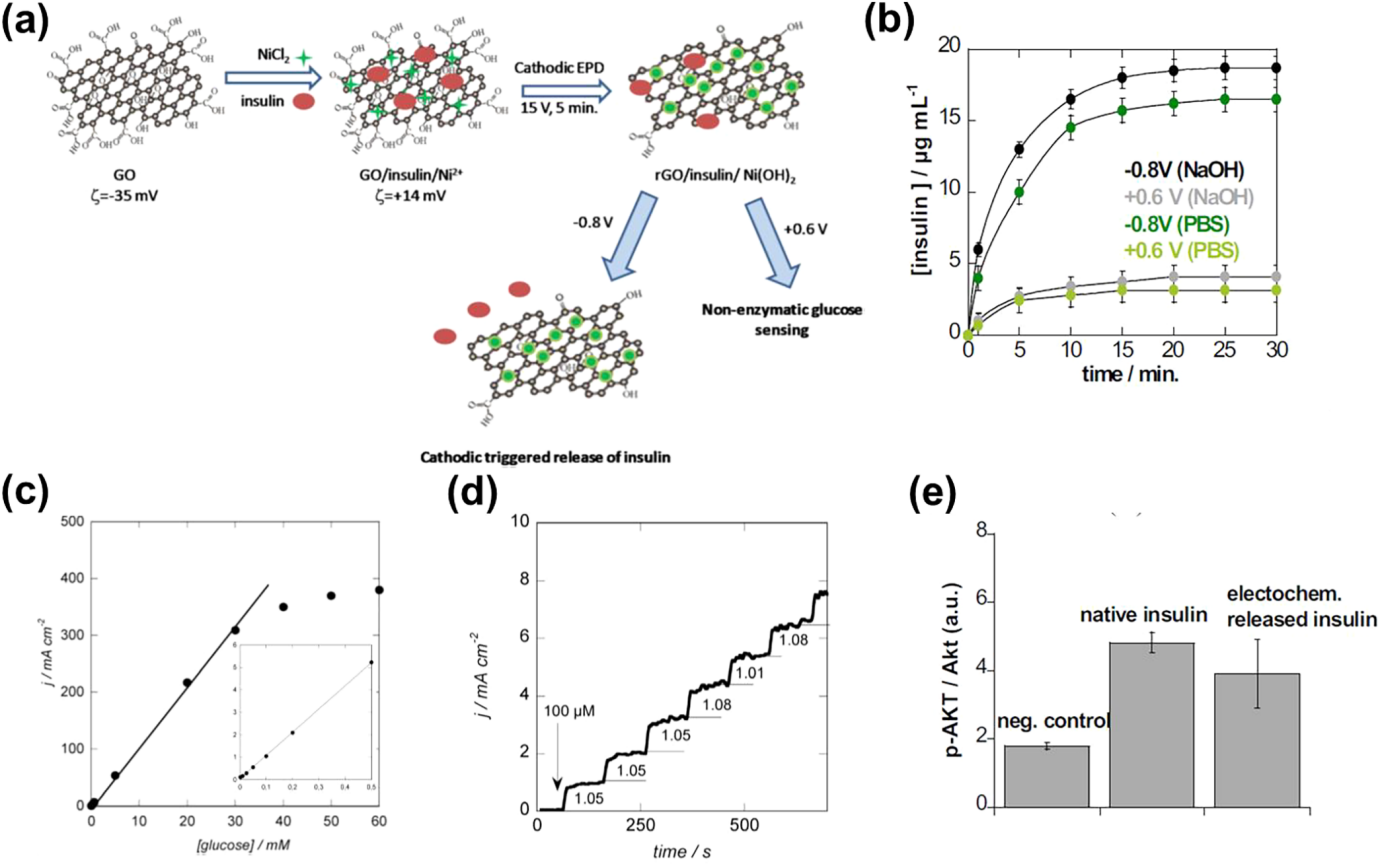
(a) Sketch describing the procedure used to prepare rGO/insulin/Ni­(OH)_2_ and to release insulin from it (Reproduced with permission from ref^65^. Copyright 2016, Elsevier). (b) Release profiles of insulin from rGO/insulin/Ni­(OH)_2_/Au into phosphate-buffered saline (PBS) (pH 7.4) and NaOH (0.1 M) aqueous solution upon application of potential pulses at −0.8 and +0.6 V (Reproduced with permission from ref^65^. Copyright 2016, Elsevier). (c) Calibration curve for rGO/insulin/Ni­(OH)_2_/Au (inset: zoom to lower concentrations) (Reproduced with permission from ref^65^. Copyright 2016, Elsevier). (d) Amperometric response curve of rGO/insulin/Ni­(OH)_2_/Au at +0.6 V vs Ag|AgCl with successive additions of glucose up to a total of 1 mM (Reproduced with permission from ref^65^. Copyright 2016, Elsevier). (e) Insulin activity expressed as the ratio between active protein kinase B phosphorylation (p-Akt) and nonactivated Akt (a.u.) for native insulin and electrochemically released insulin in comparison to a negative control (CTL neg.), which corresponds to cells without insulin treatment (Reproduced with permission from ref^65^. Copyright 2016, Elsevier).

Moreover, rGO/insulin/Ni­(OH)_2_/Au electrodes were multifunctional since, in a previous study, the same group reported that the rGO/Ni­(OH)_2_ composite exhibits very high electrocatalytic activity toward glucose oxidation in alkaline environments.^66^ Thus, rGO/Ni­(OH)_2_ displayed a linear current response for glucose concentrations ranging from 15 μM to 30 mM with a limit of detection and an analytical sensitivity of 15 μM and 11.4 ± 0.5 mA/(cm^2^·mM), respectively (Figure 2c).^65^ The detection of glucose was performed by chronoamperometry in an alkaline solution (0.1 M) by applying a potential of +0.6 V. The rGO/Ni­(OH)_2_/Au electrode showed a rapid sensing response time and high stability, maintaining performance with minimal degradation over repeated use and long-term storage. Moreover, it exhibited excellent specificity for glucose, with negligible interference from common substances like ascorbic acid, uric acid, and dopamine (Figure 2d).^65,66^ Interestingly, no insulin burst delivery from rGO/insulin/Ni­(OH)_2_/Au occurred at the glucose-sensing potential (Figure 2b), while the metabolic activity of released insulin from the matrix was preserved, regardless of the applied potential (Figure 2e).^65^ The integration of the two functionalities (*i.e*. insulin release at −0.8 V^67^ and glucose oxidation detection at +0.6 V^66^) in a single-step process,^65^ which took advantage of the excellent electrical conductivity of rGO and the electrocatalytic properties of Ni­(OH)_2_, offered a very competitive approach for therapeutic applications. Unfortunately, it should be mentioned that The Royal Society of Chemistry recently published an expression of concern^68^ in order to alert readers that preoccupation had been raised regarding the reliability of the data reported in ref 66.

In another work, Shi and co-workers developed a multilayered system formed by a hybrid hydrogel deposited onto a titanium sheet.^69^ More specifically, the hydrogel was obtained by simultaneously electrodepositing (*i.e*. in a single-step process) chitosan (CS) and layered double hydroxides (LDHs) onto the titanium electrode (Figure 3a). For this purpose, a dispersion was prepared by adding hexagonal-shaped LDHs (Figure 3b), which were previously synthesized by the hydrothermal method using Mg_2_Al­(OH)_6_Cl·*x*H_2_O, to a CS solution. Before the electrodeposition, insulin was successfully loaded into the LDHs, which showed a loading capacity of 20.3%.^69^ Interestingly, the ζ potential in neutral water of LDHs changed from +8.6 to −4.2 mV after the loading of the protein. This change of sign was attributed to a charge compensation effect since the negatively charged residues of insulin (*e.g*. Asp and Glu) resulted in a ζ potential of −19 mV for the protein alone, which evidenced the electrostatic affinity of insulin molecules toward the surface of LDHs. On the other hand, the release of the loaded insulin from the hybrid hydrogel was stimulated by anions, pH, and electrical signals.^68^ The type and concentration of anions, as well as the pH, affected the electrostatic attraction between insulin molecules and the surface of LDHs, altering not only the amount of released insulin but also the release rate (Figure 3c,d). Also, the release profile was significantly accelerated by imposing electrical signals (Figure 3e), with positive potentials having more influence than negative potentials. Indeed, the authors proposed a finely tuned insulin release by precisely adjusting the electrical signal to a switching positive voltage in the on–off mode for 30-min periods (Figure 3f). In a subsequent study,^70^ the preparation of the insulin-loaded CS-LDHs hybrid hydrogel was scaled up, while the electroregulated release of insulin was demonstrated both *in vitro* and *in vivo* using diabetic rats. The reduction of the glucose level obtained by applying an on–off electrical signal was achieved without causing damage to the tissues (Figure 3g), which proved the potential application of these hybrid hydrogels.^70^

**Figure 3:**
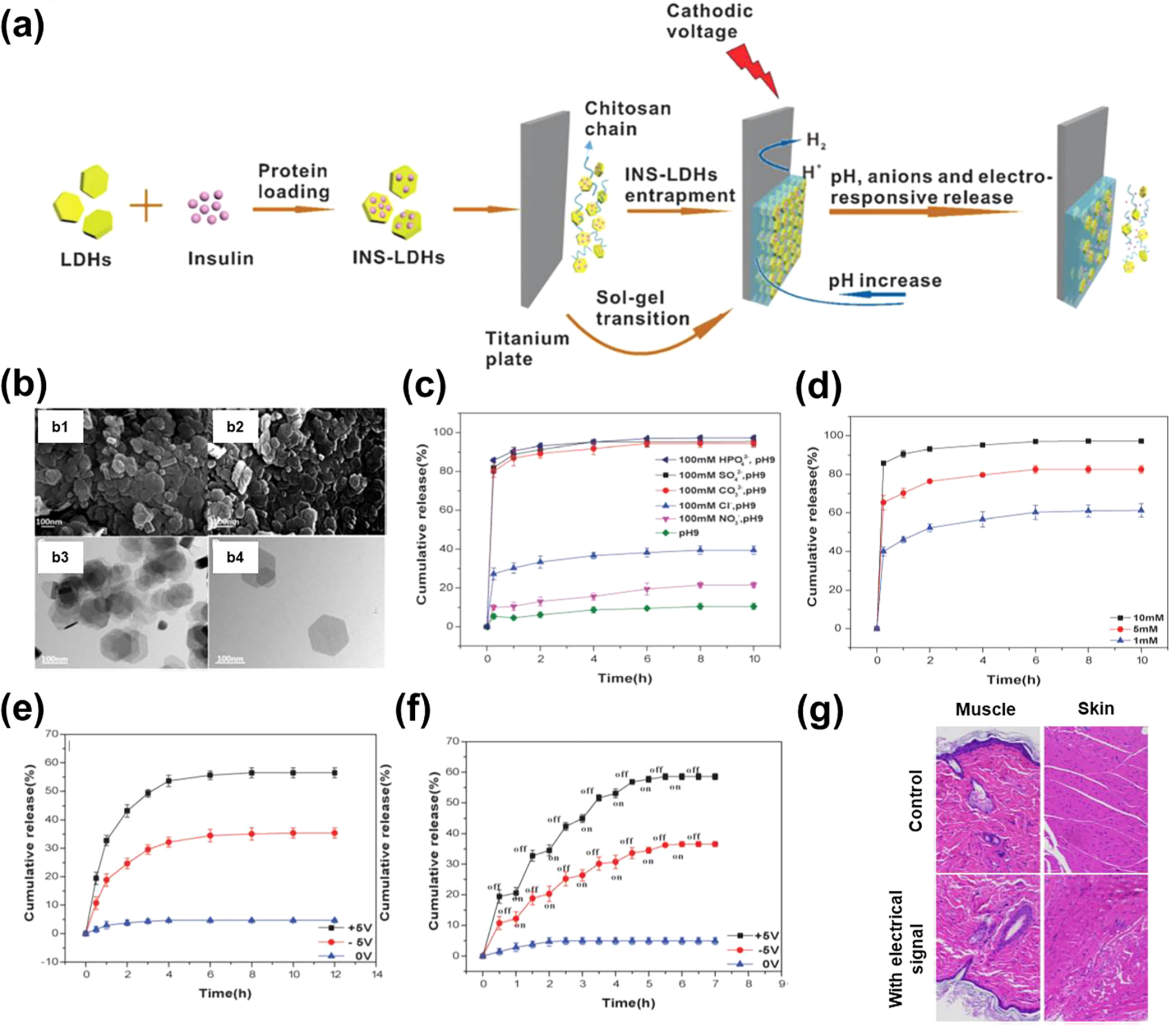
(a) Sketch describing the procedure used to prepare insulin-loaded CS-LDHs hybrid hydrogels to promote insulin release (Reproduced with permission from ref^69^. Copyright 2015, The Royal Society of Chemistry). (b) SEM (b1-b2) and TEM (b3-b4) images of the CS-LDHs hydrogel (Reproduced with permission from ref^69^. Copyright 2015, The Royal Society of Chemistry). (c–f) Cumulative release profiles of insulin from the insulin-loaded hybrid hydrogel in presence of: (c) different ions at pH 9, (d) different concentration of ions (10, 5 and 1 mM in PBS at pH 7.4), (e) electrical signals at a given potential, and (f) electrical signals as on-of mode (Reproduced with permission from ref^69^. Copyright 2015, The Royal Society of Chemistry). (g) Stained muscle and skin after the electroregulated release of insulin (Adapted with permission from ref^70^. Copyright 2022, Wiley).

On the other hand, Zare and co-workers^71^ reported an electroresponsive insulin delivery system that operated at low voltages. This multilayered system was constituted by an insulin-loaded layer of poly­(methyl methacrylate*-co*-methacrylic acid) (PMMA) copolymer protected by a CS layer, which was both deposited on a gold electrode. The voltage applied to the electrode induced the reduction of water, causing a change in the pH of the medium that dissolved the PMMA layer and released the insulin, which had been previously loaded with an efficiency of 32%. The CS layer did not hinder the release of insulin but avoided the delamination of the PMMA layer when the voltage was applied. After examining the effect of different voltages (from −0.5 to −1.5 V) and currents (from −50 to −300 μA) on the electrostimulated release, 82% of insulin was released under the optimal conditions, which corresponded to pulses of −1.5 V for 20 s. Such a value was significantly higher than the 6% achieved by passive diffusion. Additionally, the electrostimulated release of other drugs (*e.g*. fluorescein, meloxicam, and curcumin) was tested to show the general performance of this PMMA-based system as a potential carrier.^71^

## Transdermal Insulin Delivery Systems

Transdermal drug delivery systems (TDDS) offer a minimally invasive method for administering medications through the skin, providing controlled and sustained release of drugs into the bloodstream.^72–76^ These devices have achieved significant advances in real-time glycemic control, offering a dynamic and effective option for maintaining blood glucose levels. TDDS typically consists of a patch that adheres to the skin and contains the drug within a polymer matrix, allowing for consistent and prolonged administration. The advantages of TDDS include improved patient compliance because of the ease of application, reduced frequency of administration, minimization of side effects due to stable drug levels, and elimination of needles and injections. Recently, researchers have developed transdermal patches that incorporate microneedles or permeation enhancers to facilitate insulin delivery through the skin, demonstrating their potential to maintain stable blood glucose levels and improve the quality of life of diabetic patients.

Microneedle array patches (MAPs) represent a painless, minimally invasive alternative to traditional subcutaneous injections used for drug delivery.^77–79^ The microneedles of such patches puncture the stratum corneum to access the epidermal and dermal layers of skin without injuring the capillaries and subcutaneous nerves, allowing drugs (including insulin) to be delivered directly into the bloodstream. In addition to enabling local cargo delivery and targeted therapeutic delivery, MAPs allow for the self-administration of therapeutics without the need for trained professionals. Furthermore, MAP technologies are very attractive due to their durability, scalability, and cost-effectiveness. The advantages and limitations of transdermal MAPs have been recently reviewed, considering factors such as the principles of transdermal penetration (enough to penetrate and overcome the viscoelastic forces of skin), drug delivery efficiency, research progress, synergistic innovations among different methods, patient compliance, skin damage, and post-treatment skin recovery.^80^ In the particular case of insulin, which is a macromolecular drug, an important advantage of transdermal insulin delivery technologies is the diameter of microneedles (from 500 to 800 μm), that is much smaller than the diameter of insulin pump needles (0.5 mm).^81^ In recent years, significant efforts have been made to engineer insulin-loaded MAPs, even though its release has largely been controlled by biological signals (*e.g*. the presence of hydrogen peroxide and reactive oxygen species) instead of electrical stimuli.^82^

Iontophoresis consists on the utilization of weak electric currents (<0.5 mA/cm^2^) to promote the transdermal delivery of hydrophilic and charged drugs without damaging the skin.^83^ In the case of macromolecular drugs, iontophoresis enhances the release from MAPs by increasing the guided diffusion via electromigration and the skin permeability via electro-osmosis.^84^ In addition, iontophoresis has been found to improve drug localization and targeting, as well as to reduce systemic side effects by controlling the rate and depth of drug penetration.^85–88^

In a pioneering work, Garland et al.^89^ combined the utilization of iontophoresis and drug-loaded polymeric microneedles for controlled transdermal insulin delivery. MAPs made of a copolymer of methylvinylether and maleic anhydride (PMVE/MAH) were prepared and, subsequently, optimized by examining the influence of both the microneedle height and microneedle density on the delivery of small hydrophilic drugs (Figure 4a). Interestingly, all the hydrophilic compounds showed a loading efficiency higher than 90%, while for the optimum array, the release efficiency ranged from 63 to 82%, depending on the drug, within a period of 6 h, independently of the application or not of iontophoresis. Finally, the patches with the optimized PMVE/MAH microneedle array design, which displayed a microneedle height of 600 μm and a density of 361 microneedles per cm^2^ (Figure 4a[Fig fig4]), were used to evaluate the electroregulated release of insulin.^89^ In this case, the insulin stored in the base plate remained undelivered due to clogging in the absence of an electric current. Indeed, the release efficiency at 6 h was 4.85% only. When iontophoresis was applied, the release efficiency increased to 9.87%, which indicated that the electric current facilitated the transport of insulin from the base plate to the skin.

**Figure 4:**
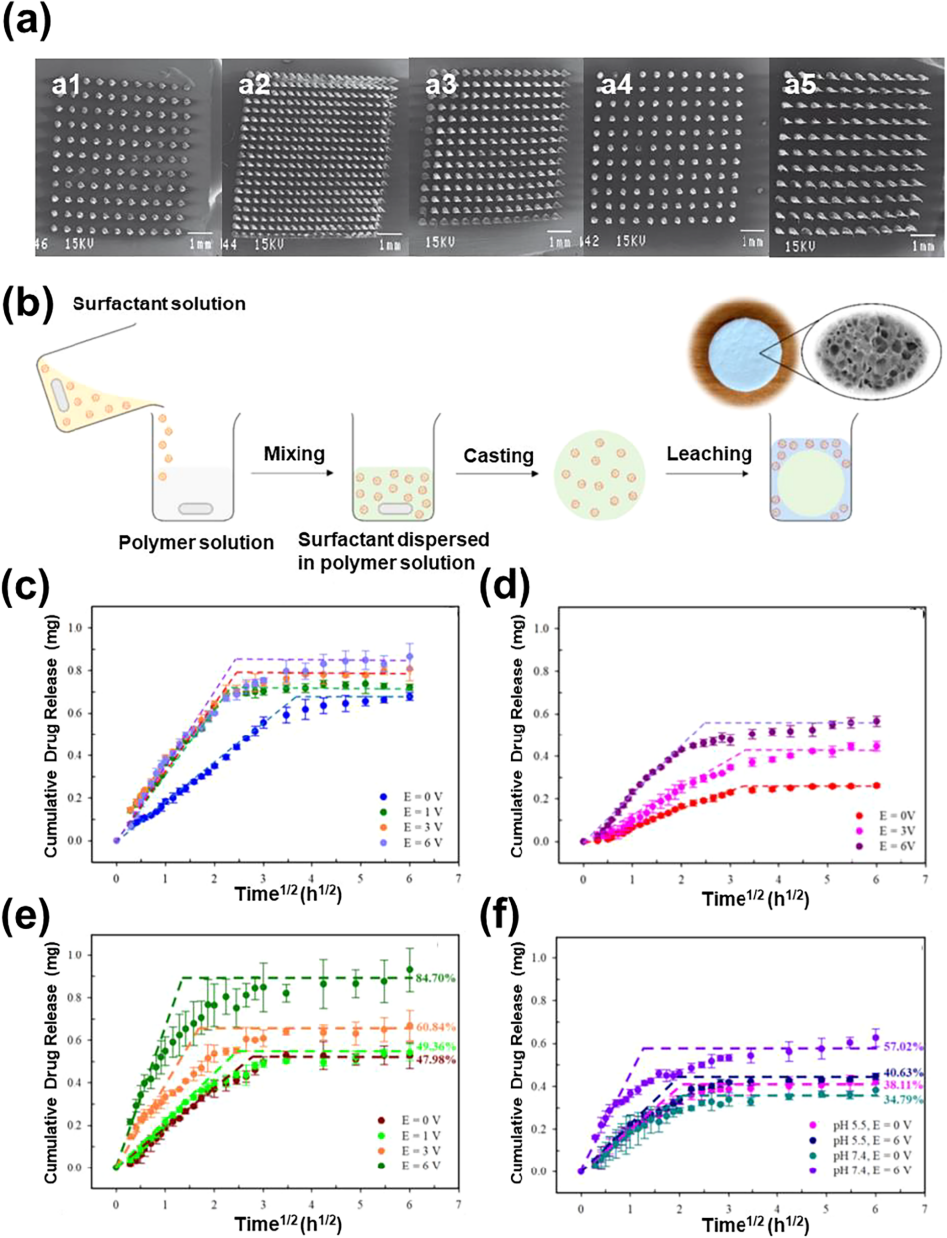
(a) SEM images of PMVE/MAMN microneedle arrays with different microneedle heights (*h*) and microneedle interspacings (*L*): (1) *h* = 350 μm, *L* = 300 μm; (2) *h* = 600 μm, *L* = 50 μm; (3) *h* = 600 μm, *L* = 150 μm; (4) *h* = 600 μm, *L* = 300 μm; and (5) *h* = 900 μm, *L* = 300 μm. (Adapted with permission from ref^89^. Copyright 2012, Elsevier). (b) Sketch displaying the procedure used to prepare porous TPU scaffolds (Reproduced with permission from ref^90^. Copyright 2022, Elsevier). (c) Insulin release and (d) *in vitro* insulin skin permeation under the effect of electric voltages as a function of time^1/2^ using porous insulin/TPU scaffolds (Adapted with permission from ref^90^. Copyright 2022, Elsevier). (e) Insulin release and (f) *in vitro* insulin skin permeation under the effect of electric voltages versus time^1/2^ using insulin/PAni:PSS/TPU scaffolds (Adapted with permission from ref^92^. Copyright 2024, Springer).

Other materials have also been studied for fabricating transdermal patches. Sirivat and co-workers^90^ developed porous thermoplastic polyurethane (TPU) scaffolds for insulin transdermal delivery using solvent casting and a particulate leaching technique with a porogen (Figure 4b). The choice of TPU, which is a fully thermoplastic elastomer (*i.e*. it becomes flexible when heated and hard when cooled), was made because of the following advantages: high stretch ability, high tear resistance, good chemical resistance, nontoxicity to human skin, and its potential to be recycled after usage.^91^ The interconnected porous structure of the matrix resulted in the rapid absorption of insulin and its efficient release. Furthermore, it was observed that both the release rate and the diffusion coefficient of insulin increased with the pore size and the electric voltage. Obviously, the diffusion of insulin molecules was easier with the increasing size of the pathway, and the amount of released insulin changed from 38.1 to 61.7% when the percentage of porogen used in the manufacturing process augmented from 20 to 30% v/v.^90^ Regarding the electric voltage and considering the highest pore size, the release of loaded insulin increased from 61.7% in the absence of voltage, to 65.6, 73.5, and 78.7% for voltages of 1, 3, and 6 V, respectively (Figure 4c).^90^ Thus, the electric voltage affected the insulin release by inducing an electrorepulsive force, which drove the ionized insulin molecules out of the matrix more efficiently. The potential of the porous TPU as an inulin transdermal patch under iontophoresis was confirmed by conducting *in vitro* skin permeation experiments with pig skin (Figure 4d).

In another work, the same authors improved the electrical response and, consequently, the insulin delivery capability of porous TPU by adding polyaniline (PAni) doped with poly­(4-styrenesulfonic acid) (PSS).^92^ The insulin-loaded PAni:PSS, which was prepared by mixing a homogeneous PAni:PSS aqueous solution with an insulin NaHCO_3_ solution, was simply drop-cast onto the surface of porous TPU. The resulting system, insulin/PAni:PSS/TPU, was noncytotoxic to human skin. The transdermal delivery of insulin, which was electrostatically bonded to PAni:PSS, increased to almost 75% when an electric voltage of 6 V was applied for 2 h (Figure 4e), evidencing that PAni:PSS is an effective insulin carrier that enhances the release and shortens the time for that.^92^ Similarly, *ex vivo* release-skin permeation studies revealed that the electroregulated drug release was more efficient with insulin/PAni:PSS/TPU than insulin/TPU devices, especially at neutral pH (Figure 4f).^92^

Yang et al.^93^ reported an innovative MAP for electroregulating the release of insulin, which was able to retract automatically the microneedle array from the previously created microholes in the skin (Figure 5a–e). This property minimized the risk of skin allergy by preventing microneedles from remaining in the skin for a long time. The iontophoresis MAP was powered by a smartphone, allowing us to control the dosage of insulin by regulating the current intensity. In order to maximize the iontophoretic process, insulin was loaded into positively charged nanovesicles (Figure 5f). Thus, previous studies showed that the utilization of positively charged nanovesicles as nanocarriers of the negatively charged insulin enhanced the transdermal drug penetration, which was attributed to the active electro-osmosis and the electrostatic interaction between the skin and the nanovesicles.^94^ Conversely, negatively charged nanovesicles were found to be disadvantageous to the penetration of insulin because of the reverse electro-osmosis.^94^ The effectiveness of such a transdermal delivery system was demonstrated both *in vitro* and *in vivo*.^93^ In particular, rat model studies evidenced that the utilization of MAPs coupled to iontophoresis and positively charged nanovesicles was very effective in regulating the blood glucose level, avoiding hypoglycemia (Figure 5g).

**Figure 5:**
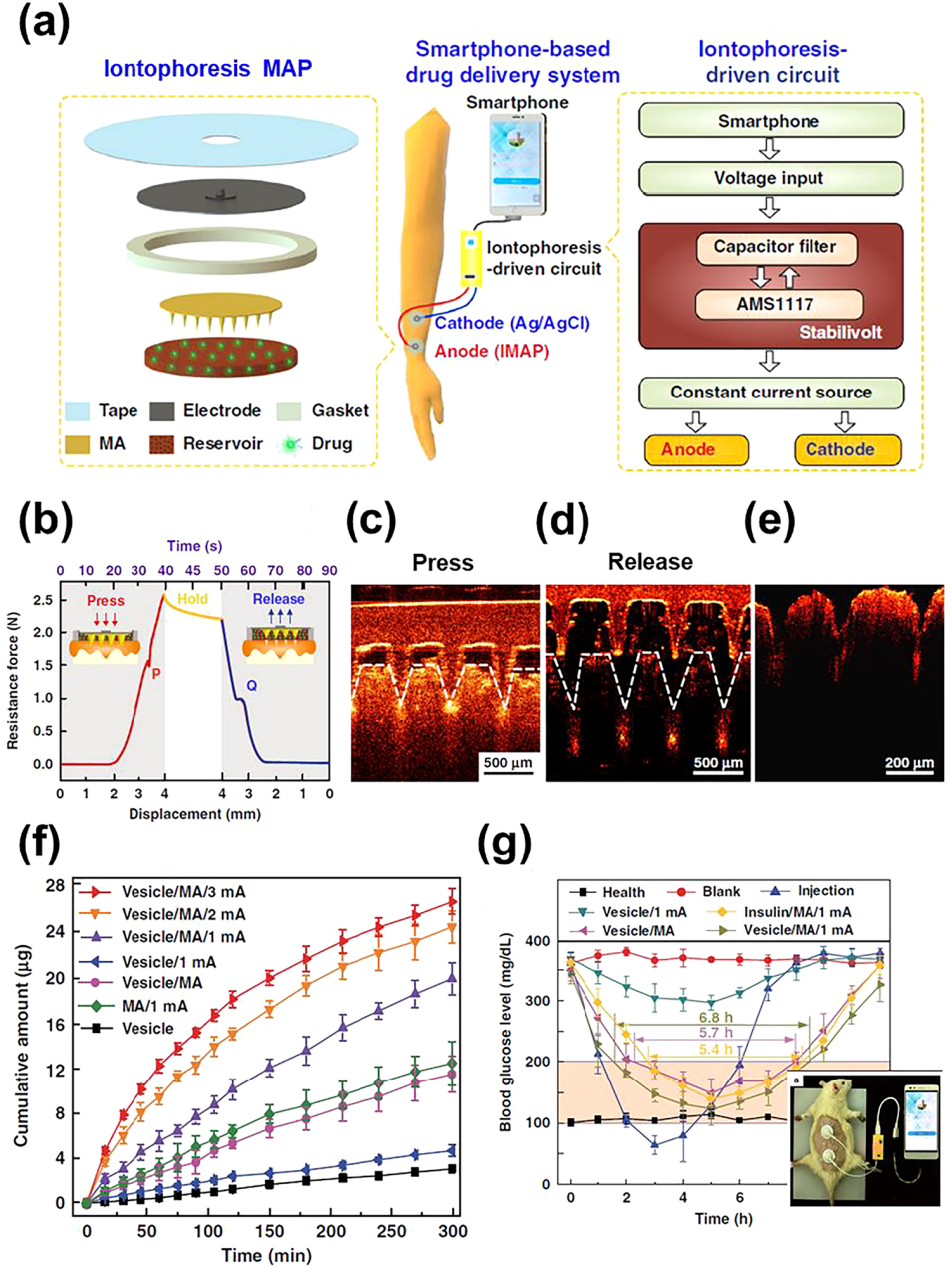
(a) Sketch of the smartphone-powered transdermal insulin release system (center). The iontophoresis MAP is displayed on the left, while the iontophoretic-driven circuit is shown on the right. The iontophoresis MAP consists of a medical tape, a conductive film working as the electrode, an antiseepage gasket, a microneedle array, and a reservoir loaded with insulin solution (Reproduced with permission from ref^93^. Copyright 2020, Nature). (b) Mechanical response curves of the iontophoresis MAP during the press-release assays (Reproduced with permission from ref^93^. Copyright 2020, Nature). (c–e) Optical coherence tomography images of skin penetration by the microneedle array during the press (c), retraction of the microneedle array during the release (d), and poked skin after press-release assay (e) (Adapted with permission from ref^93^. Copyright 2020, Nature). (f) Cumulative permeation amount of insulin using vesicles stimulated with different current densities (Adapted with permission from ref^93^. Copyright 2020, Nature). (g) Blood glucose level of diabetic rats treated using different transdermal insulin delivery approaches, including iontophoresis with insulin-loaded vesicles. The inset shows the coupling of the smartphone-powered iontophoresis device to a diabetic rat transcutaneously treated (Adapted with permission from ref^93^. Copyright 2020, Nature).

Based on their previously reported multilayered electrode for electroregulated release of insulin,^65^ Szunerits and co-workers developed a novel approach by integrating nanoheaters into insulin-loaded transdermal patches.^95^ The nanoheaters, which consisted of nanoperforated gold thin layers generated by colloidal lithography, exhibited outstanding electrothermal properties (*i.e*. a steady state temperature of 50 °C was reached in a few seconds applying a power density below 250 mW/cm^2^).^96^ rGO films were deposited on the gold thin layer to construct the electrothermal patch, which was subsequently loaded with insulin by drop-casting the drug onto the rGO side. Insulin was loaded in the rGO layer with an efficiency of 95% due to the formation of noncovalent bonds,^95^ such as hydrogen bonding, π–π stacking, and electrostatic interactions.^97^ The release of insulin from the electrothermal patch was achieved at physiological temperature upon the application of a dc electrical bias of 1 V (*i.e*. the biological activity of insulin was preserved when the applied electrical bias was lower than 1.6 V). The patch heated with the applied voltage, reaching a steady temperature of around 52 °C, which triggered the release of insulin. Furthermore, the formation of micropores was promoted at such a temperature, favoring the delivery across the stratum corneum, the outermost layer of the skin. Nevertheless, as occurred for other studies reported by Szunerits,^68^ The Royal Society of Chemistry recently published an expression of concern in order to warn readers regarding the reliability of the data reported for this electrothermal patch.^98^

Tari and co-workers^99^ examined the potential of water-soluble conducting polymer NPs (in this case, PPy) for controlled delivery of insulin using iontophoresis. PPy NPs were prepared by emulsion polymerization using sodium dodecyl sulfate (SDS) as both dopant and stabilizing agent, as was previously reported for other conducting polymers.^100,101^ The loading of insulin, which was performed at neutral pH,^99^ occurred through the electrostatic interactions between the positively charged drug and the SDS anions. The transdermal delivery was studied by using both anodal and cathodal iontophoresis, showing low (20.48 μg/cm^2^) and high (68.2 μg/cm^2^) insulin permeation, respectively, for an electrical stimulation of 60 min at 0.13 mA/cm^2^. When the conditions (*i.e*. pH, formulation, current, etc.) used for the cathodal iontophoresis-driven insulin permeation were optimized, the cumulative transdermal transfer over 48 h reached 834 μg/cm^2^.^99^

Recent advances in MAPs have incorporated glucose detection to exploit the potential of developing an automated delivery system triggered by glucose levels. This concept simplifies the complexity of diabetes treatment by integrating glucose monitoring and insulin release into a single device.^102–107^ Devices able to link the glucose sensor output to insulin delivery through a control algorithm are denoted closed-loop systems.^108^ However, in general, electrical stimulation does not participate in such self-regulated release devices. For example, Kim and co-workers^102^ reported a closed-loop system capable of delivering insulin in response to the blood glucose level. For this purpose, stainless steel microneedle arrays were coated with a porous thin layer of poly­(lactic-*co*-glycolic acid) (PLGA), and the PLGA pores were filled with insulin, sodium bicarbonate, and glucose oxidase (GOx). Then, the pores were coated with another thin PLGA layer to prevent the encapsulated insulin from being delivered in response to specific glucose concentrations. While sodium bicarbonate was employed as a pH-sensitive compound, GOx was a glucose-specific enzyme used to oxidize glucose, which diffused across the top PLGA layer into the pores, converting it into gluconic acid and thereby lowering the local pH The resulting protons reacted with sodium bicarbonate and formed CO_2_ that, when the glucose concentration is very high, creates pressure inside the pores breaking them and thereby promoting the release of insulin. This glucose-responsive device was successfully proven *in vitro* using porcine skin and *in vivo* with diabetic rats.

Other examples have described similar closed-loop systems but using different recognition and responsive elements for the detection of glucose and the delivery of insulin, respectively, although without the aid of electrical signals.^103–106^ Gu and co-workers combined insulin-loaded microneedles with phenylboronic acid (PBA)-containing polymeric matrix, which was responsive to the presence of glucose, forming glucose–boronate complexes and promoting the release of insulin.^103^ Wang et al.^104^ reported a similar microneedle patch based on PBA-modified CS particles and poly­(vinyl alcohol) (PVA)/poly­(vinylpyrrolidone) (PVP) hydrogel for efficient insulin delivery in response to glucose concentration. Ali et al.^105^ developed a closed-loop insulin delivery system using hybrid hydrogels that were prepared by cross-linking PVA and CS NPs using formylphenylboronic acid as a cross-linker. Additionally, Ma and co-workers^106^ engineered an insulin-loaded glucose-responsive cannula capable of rapidly releasing insulin under hyperglycemic conditions using a hydrogel functionalized with fluorophenylboronic acid (FBPA), which enabled the binding of glucose. Among other advances in glucose monitoring for diabetes management, the principles of electrochemical sensing of glucose as an important part of closed-loop systems were reviewed in detail by Ma et al.^107^

In a very recent study, electrical signals were incorporated into such dual sensing/release devices as part of the glucose monitoring system (*i.e*. using an electric current to detect the oxidation of glucose).^108^ More specifically, Huang et al.^109^ conceived a sophisticated integrated electronic/fluidic microneedle patch for both glucose monitoring using an electrochemical sensor and insulin delivery. Microneedles were prepared by using hollow stainless steel tubes, whose inner walls were subsequently coated with electrodeposited gold and PMMA to improve the electrochemical properties of the steel and insulate the electrodes from solution interference, respectively. Subsequently, customized polyimide electrodes with silver wires attached to the welding electrodes were incorporated into the resulting microneedle array. The electrodes for glucose-sensing were fabricated by incorporating a conducting layer of electrodeposited single-wall carbon nanotubes from an adhesive Nafion solution. Finally, GOx was immobilized on the surface, creating a cross-linked mesh with glutaric dialdehyde. The function of the resulting sensing microneedle array (Figure 6a) was the transdermal detection of glucose through hydrogen peroxide production using a small electrical voltage (∼0.5 V). Then, an insulin delivery module with microchannels, a fluidic pump, and embedded circuits was coupled to the same hollow microneedle sensing array (Figure 6b), resulting in a wearable integrated system for glucose sensing and insulin delivery using wireless communication functions (Figure 6c).^109^

**Figure 6:**
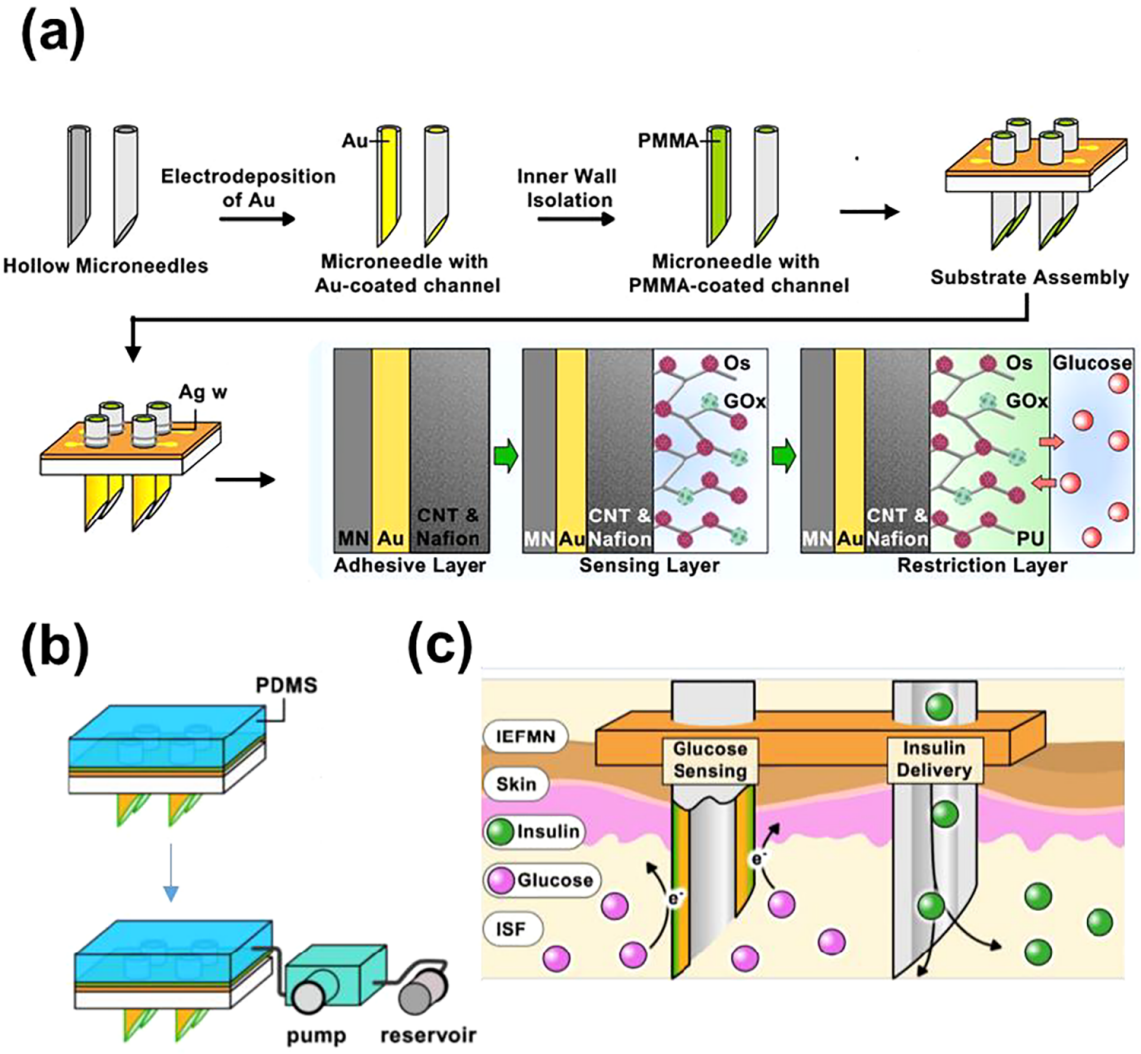
(a) Preparation of glucose-sensing microneedle arrays (Adapted with permission from ref^109^. Copyright 2024, Ivyspring International Publisher). (b) Scheme of the insulin delivery module (Adapted with permission from ref^109^. Copyright 2024, Ivyspring International Publisher). (c) Illustrations depicting the operational mechanism of the integrated electronic/fluidic microneedles (IEFMN) patch for transdermal glucose detection and drug delivery (Adapted with permission from ref^109^. Copyright 2024, Ivyspring International Publisher).

The first electrostimulated sensing/release iontophoretic device for diabetic patients was proposed around ten years ago by Kim and co-workers.^110^ Those authors prepared a wearable patch for sweat-based diabetes monitoring and therapy units (Figure 7a). The sensing unit was composed of humidity, glucose, pH, and tremor sensors, while the therapeutic unit consisted of microneedles, a heater, and a temperature sensor. The electrochemical sensors that monitored the aforementioned biomarkers were fabricated by using graphene doped with gold and combined with a gold mesh (Figure 7b). The microneedles were made of a bioresorbable polymer (PVP) coated with a phase-change material (Figure 7c) able to release the drug into the bloodstream once a programmed temperature threshold is reached. More specifically, when the amount of sweat (monitored through the humidity sensor) exceeded a critical threshold, glucose monitoring is activated and corrected by simultaneously measuring pH and temperature. If the detected glucose concentration is excessive, the heaters integrated into the therapeutic unit are activated, dissolving the phase-change material and releasing insulin from the bioresorbable microneedles.

**Figure 7:**
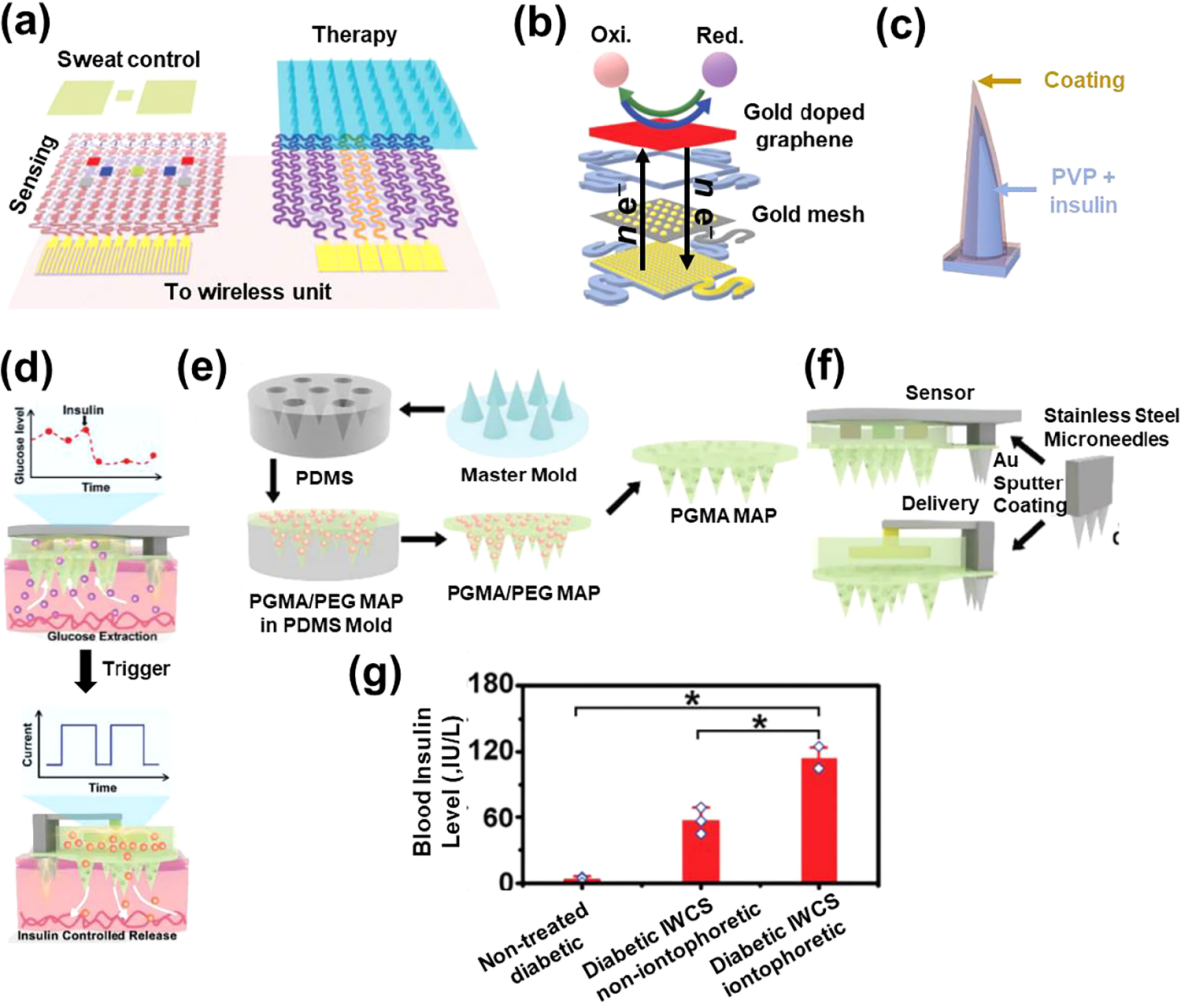
(a) Sketch of the diabetes patch, which is composed of the sweat-control, sensing, and therapy units (Adapted with permission from ref^110^. Copyright 2016, Nature). (b) Sketch of the electrochemical sensing unit (Adapted with permission from ref^110^. Copyright 2016, Nature). (c) Sketch of the bioresorbable microneedles (Adapted with permission from ref^110^. Copyright 2016, Nature). (d) Illustration of the IWCS for glucose monitoring and electrically triggered insulin delivery (Adapted with permission from ref^111^. Copyright 2021, Wiley). (e) Process used to prepare the PGMA-based MAPs employed in IWCS (Adapted with permission from ref^111^. Copyright 2021, Wiley). (f) Sketches showing the components of the sensing and delivery devices in the IWCS system (Adapted with permission from ref^111^. Copyright 2021, Wiley). (g) Plasma insulin levels in diabetic rats treated with the IWCS and the noniontophoretic IWCS device for 2 h (Adapted with permission from ref^111^. Copyright 2021, Wiley).

More recently, Xie and co-workers^111^ took advantage of electrical voltages for finely tuning insulin delivery as an automated response to glucose detection. Such a smart, integrated, wearable closed-loop system (IWCS) combined continuous glucose monitoring from interstitial fluid with insulin delivery in mesoporous microneedle array technology. Thus, the developed MAPs allowed pain-free penetration of the stratum corneum for glucose extraction and insulin administration (Figure 7d). Mesoporous MAPs were obtained by centrifuging a mixture of poly­(glycidyl methacrylate) (PGMA) and polyethylene glycol (PEG), which acted as a porogen, in methoxyethanol in a polydimethylsiloxane (PDMS) mold. After cross-linking the PGMA under UV light, the matrix was removed from the mold, and the porogen was dissolved in an ethanol: water mixture (Figure 7e). The glucose monitoring sensor (Figure 7f) consisted of MAPs for accessing the interstitial fluid, a reverse-iontophoretic glucose extraction system with the gold-coated stainless steel microneedles, and a planar three-electrode system (*i.e*. an enzymatically functionalized carbon electrode as working electrode, a platinum-coated carbon electrode as counter electrode, and an Ag|AgCl reference electrode).^111^ The IWCS device also contained a sophisticated feedback mechanism designed to trigger the release of insulin only when glucose levels exceeded a certain threshold. More specifically, the conductive microneedle array applied low-voltage electrical pulses to an insulin-loaded mesoporous membrane, causing expansion of the pores at the membrane and allowing insulin to flow and enter the skin. The application of electrical pulses enabled the release of small amounts of insulin, mimicking physiological insulin secretion. *In vivo* studies with diabetic rat models demonstrated that the glucose concentration treated with the iontophoresis system dropped to normoglycemia within 1.5 h and remained stable. In contrast, diabetic rats treated with subcutaneous insulin injections experienced a rapid drop in glucose followed by a quick rebound to hyperglycemia.^111^

Although most MAPs rely on iontophoresis or electro-osmosis to deliver drugs, a novel approach was proposed by Lu and co-workers in a recent study.^112^ Those authors used electrical stimulation to induce the swelling of thiolated silk fibroin microneedles for insulin delivery. Microneedles were designed to be electroresponsive, altering their swelling capacity through the reversible formation (unenergized oxidation state) and breaking (energized reduction state) of disulfide bonds in the silk fibroin matrix. Such oxidation and reduction changes in the disulfide bonds, which are reversible and typically observed in thiolated materials,^113,114^ were supported by different structural characterization studies. Graphene was added to enhance the electrical response of thiolated silk fibroin microneedles, contributing to regulating the release rate of insulin through better control of the swelling degree. The swelling ratio of thiolated silk fibroin microneedles increased from 72% without electrostimulation to more than 200% with electrostimulation.^112^ *In vivo* tests on diabetic rats confirmed that thiolated silk fibroin microneedles modified with graphene effectively controlled blood glucose levels, with insulin release increasing upon electrostimulation and decreasing when the power was off (*i.e*. the bonds reformed in the absence of electrical stimuli, reducing the swelling and slowing the release). Overall, this system allowed for controlled insulin delivery in response to blood glucose levels, offering a potential method for managing diabetes by synchronizing insulin release with real-time glucose monitoring.^112^

Despite MAPs representing a stride in insulin delivery, these systems currently still have notable disadvantages, including potential skin irritation or allergic reactions, the inability to deliver large molecules (like insulin) efficiently through the skin barrier, and variability in absorption rates due to differences in skin types and conditions. These challenges must be addressed to fully achieve the potential of TDDS for insulin delivery.

## Electrically Triggered Insulin Delivery from Injectable Systems

In the past decade, physically or chemically cross-linked injectable hydrogels have received considerable attention in the biomedical field,^115–123^ especially as DDSs.^119–123^ The outstanding behavior of hydrogels as DDS can be attributed not only to their similarity to the native extracellular matrix but also to their biocompatibility and ease of modification.^124–127^ Thus, hydrogel tuning sometimes allows for controlled release of the encapsulated drugs, endogenous (*e.g*. pH and temperature) or exogenous (*e.g*. electric field and light) stimuli.^124–130^ On the other hand, in a pioneering work in 1990, Sawahata et al.^131^ revealed the principles of chemomechanical shrinking and swelling of hydrogels when stimulated with an electric field. Those authors found that bioactive materials, including insulin, were successfully released from the hydrogel by alternately switching the electric field “on” and “off”. In a subsequent study, Kwon et al.^132^ confirmed that the volume of stimuli-sensitive hydrogel networks is particularly sensitive to external stimuli, which could be useful in the controlled release of drugs. Indeed, those authors achieved a controlled release of insulin and, by extension, other macromolecules using electrical stimuli.

Despite the enormous potential of electroresponsive hydrogels as DDSs, the utilization of these systems for insulin release has not been deeply studied. Fernando and co-workers^133^ examined the utilization of electroresponsive poly­(acrylic acid) (PAA) hydrogels for controlled delivery of insulin using intermittent electrical signals via matrix deformation. Insulin was entrapped in the polymeric matrix through the formation of attractive hydrogen bonding interactions (Figure 8a), which significantly changed the morphology of the hydrogel, giving place to the formation of globular structures. Interestingly, after the release of insulin by applying electrical stimulation, those globular structures disappeared (Figure 8b).^133^ After optimization, the prepared PAA hydrogels showed very good electrical responsivity, expanding or contracting as a function of the ionization state. This allowed the modulation of the insulin release rate, which was delivered up to 80% at 10 V stimulus, compared to only 20% delivery in the absence of stimulation. Unfortunately, the high electrical stimulus required by PAA hydrogels is not feasible for *in vivo* applications, which limits the utilization of such interesting electroresponsive hydrogels in lab assays.

**Figure 8:**
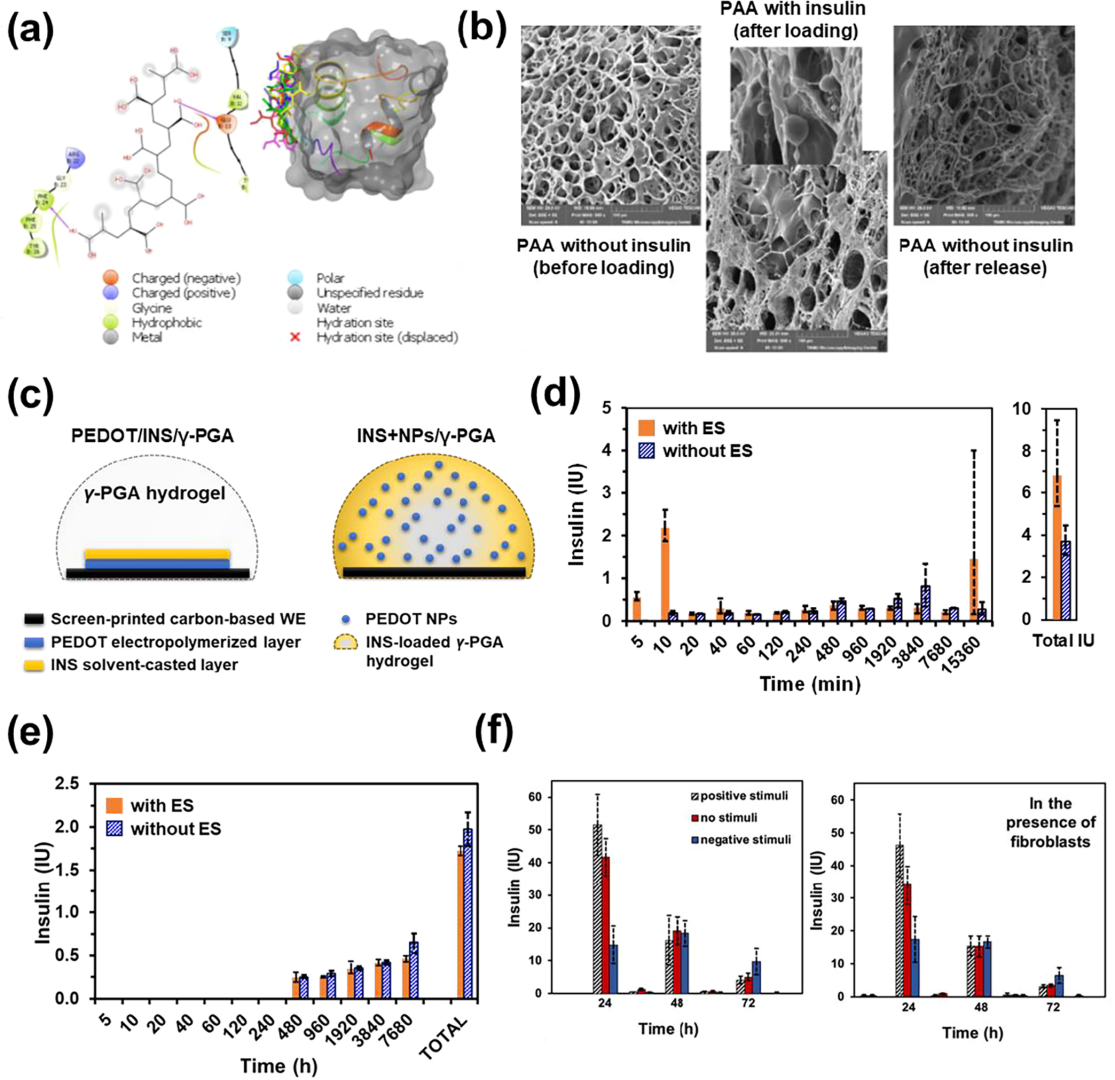
(a) Sketch showing the major binding sites of PAA for hydrogen bonding with insulin (Adapted with permission from ref^133^. Copyright 2019, Springer). (b) SEM images of the initial hydrogel (left), insulin-loaded hydrogel (middle), and hydrogel after being subjected to electrical stimulation (right). The removal of insulin after the electrical stimulus is evidenced by the reappearance of the porous structure of the hydrogel (Reproduced with permission from ref^133^. Copyright 2019, Springer). (c) Sketch of the PEDOT/INS/γ-PGA and INS+NPs/γ-PGA delivery system (Reproduced with permission from ref^134^. Copyright 2022, Elsevier). (d) Insulin release profile from PEDOT/INS/γ-PGA delivery system at specific time intervals with and without electrical stimulation. (Reproduced with permission from ref^134^. Copyright 2022, Elsevier). (e) Insulin released from INS+NPs/γ-PGA hydrogel delivery system at specific time points with and without electrochemical stimulation. (Reproduced with permission from ref^134^. Copyright 2022, Elsevier). (f) Absolute values of insulin released from the e-clickPEG-based bioplatform at specific time intervals under different electrochemical stimuli: in the lab medium (left) and in the presence of fibroblast cells (right). (Reproduced with permission from ref^146^. Copyright 2024, American Chemical Society).

In a recent study, we developed two complementary insulin delivery systems for glycemic control, which differed considerably in their pharmacokinetic profile despite being prepared using the same chemical compounds.^134^ More specifically, such two bioplatforms were based on a biohydrogel and a conducting polymer. The biohydrogel was obtained by cross-linking poly-γ-glutamic acid (γ-PGA), a biopolymer commercially relevant in cosmetics and food industry, synthesized by *Bacillus* species during fermentation,^135,136^ with cystamine. The resulting hydrogel, which is known to be noncytotoxic and biodegradable,^137–139^ has been extensively used in energy-related^140,141^ and biomedical applications.^142,143^ On the other hand, noncytotoxic and biocompatible poly­(3,4-ethylenedioxythiophene) (PEDOT),^144,145^ prepared by anodic polymerization and chemical oxidation, was the conducting polymer used for triggering the release of insulin. The first system, in which an insulin layer was sandwiched between an electropolymerized PEDOT film and the γ-PGA hydrogel (PEDOT/INS/γ-PGA in Figure 8c), displayed a rapid release response that was enhanced over a short time scale by electrical stimulation (Figure 8d). The second system, which displayed a slow and sustained insulin release (Figure 8e), was formed by the γ-PGA hydrogel loaded *in situ* with insulin and chemically synthesized PEDOT NPs (INS+NPs/γ-PGA in Figure 8c). It is worth noting that the kinetic profile of INS+NPs/γ-PGA was associated with the participation of insulin in the cross-linking reaction of γ-PGA, relating the hormone release to the degradation and loss of the hydrogel. Overall, the combination of an on-demand and fast release profile (PEDOT/INS/γ-PGA) with a sustained and slow profile (INS+NPs/γ-PGA) was expected to regulate both fast and sustained glycemic events in diabetes therapy.^134^

More recent research has been focused on addressing issues that were not fully resolved by γ-PGA-inspired designs, like, for example, an efficient kinetics release using a single platform. Thus, Muñoz-Galán et al.^146^ reported a soft platform based on thiol–yne PEG click hydrogel (e-clickPEG), which was converted into an electroresponsive incorporating PEDOT NPs, for the controlled release of insulin over an extended period of time using electrochemical stimulation. Insulin delivery was found to depend drastically on the applied voltage. More specifically, in the lab and *in vitro* assays using fibroblast cell cultures showed that insulin delivery was triggered by a positive voltage of +0.6 V, while insulin leakage was considerably reduced by a negative voltage of −0.6 V (Figure 8f).^146^ These two opposite behaviors allowed the development of the biointerface as an accurate and personalized insulin administration system, in which insulin delivery can be programmed according to real-time glucose levels. Furthermore, the utilization of the e-clickPEG hydrogel in the platform reduced the inflammation after injection because of its minimal swelling characteristics^147^ in comparison to other hydrogels.

In addition to hydrogels, conducting polymer NPs have also been proposed as injectable electroregulated insulin delivery systems.^148,149^ More specifically, Zare and co-workers loaded negatively charged insulin in PPy NPs previously prepared by microemulsion, a drug loading ratio of ∼13 wt % being achieved when a 1:1 insulin: PPy NPs solution mixture was used. Controlled and programmed insulin release was successfully accomplished in a few seconds when the interactions between the positively charged PPy chains and the insulin molecules were disrupted by applying reducing conditions (*i.e*. negative voltages or currents).^147,148^ Furthermore, when electrical stimulation was applied using pulses, a good correlation was found between the amount of released insulin and the number of electrical pulses.^149^

Although electroresponsive hydrogels have attracted enormous interest in the biomedical field, not enough attention has been given to their specific use as insulin injectable delivery systems. In fact, the number of studies developed around electroresponsive injectable systems (whether hydrogels or NPs) is anomalously scarce in relation to the number of studies in other applications. It is expected that, in the next few years, this situation will be reversed and studies will begin to emerge where the ability of these smart injectable materials as emerging theranostic platforms will be as influential as that of other materials capable of responding to other types of stimuli.^150^

## Combined Approaches

This last section is dedicated to discuss the studies in which progress has been made by combining two of the approaches described in the previous sections (*i.e*. implantable devices, transdermic liberation systems, and injectable systems), or by integrating one of such approaches with conventional drug administration procedures (*i.e*. oral and injection). This strategy, which is still in emerging phases, is very interesting, since it aims to join the successful aspects of different approaches in the most efficient and practical way. Elkhatib et al.^151^ proposed an innovative approach by combining the advantages of iontophoresis with oral insulin delivery. More specifically, the painless convenience of noninvasive oral delivery was merged with the effectiveness and minimally invasive character of ionthophoretic delivery to develop what they called “*gut ionthopheris*”. For this purpose, the permeation of orally administered insulin-loaded NPs across the gut wall was induced by applying a low electric current for short periods of time without damaging the intestinal tissues. The NPs were synthesized by ionotropic pregelation of an alginate nucleus, followed by subsequent polyelectrolyte complexation with CS.^151^ Insulin was added prior to polyelectrolyte complexation in order to achieve stabilization by electrostatic interaction between the negative charge of insulin and the positive charge of CS, resulting in a loading efficiency of approximately 83%. The cathodic iontophoresis was investigated both *in vitro* and *in vivo* using different operational conditions. The *in vitro* study required a current of 50 μA applied during the 1 h cycle, which consisted of 10 min “on” and 10 min “off”, whereas *in vivo* investigations were performed by applying a current of 40 μA current for periods of 2 min “on” and 4 min “off”.

In addition to promoting the movement of NPs, the electric current induced a temporary and reversible disruption at the intestinal membrane, opening pores that further enhanced the permeation of insulin. Furthermore, experiments in diabetic rats demonstrated that the combination of “*gut ionthopheris*” and oral insulin-loaded NPs delivery had a hypoglycemic response faster than conventional oral insulin-loaded NPs.^151^ Overall, NPs used in that approach allowed for the encapsulation of insulin, preventing its degradation in the harsh gastrointestinal environment, while the iontophoresis technique provided a means of overcoming the natural barriers to drug absorption imposed by the intestinal membrane.

In another combined approach, Gong et al.^152^ developed a closed-loop system for continuous glucose sensors and on-demand insulin delivery by iontophoresis and electrical stimulation. This was part of a wireless and wearable complex platform for diabetic wound management named Thera-patch. The combined closed-loop system was based on a smart multifunctional conductive polymer hydrogel specifically designed to heal diabetic wounds, which exhibited high drug loading capacity, light transmittance, conductivity, skin adhesion, and broad-spectrum antimicrobial properties. More specifically, the hydrogel was prepared by *in situ* gelation of polydopamine-doped PPy nanofibrils into a polyacrylamide network. The on-demand delivery of insulin, which was loaded *in situ*, was electrocontrolled by applying an iontophoretic current (*i.e*. using iontophoretic electrodes), which propelled the negatively charged insulin out of the hydrogel. This stimulus depended on the glucose concentration, which was determined at the wound by using a GOx-based electrochemical sensor. In order to understand the role of insulin delivery in that application, it should be emphasized that, in addition to reducing the glucose levels in diabetic patients, insulin also enhances wound healing by stimulating proliferation, neovascularization, and collagen deposition.^153,154^ While wound healing is beyond the scope of this review, the key contribution of Gong et al.^152^ from the perspective of electrocontrolled insulin release was the integration of conductive hydrogels with iontophoretic electrodes.

## Conclusions

Electroregulated insulin delivery systems represent a great alternative to administering controlled dosages and efficiently regulate the glucose level. This review has systematically explored the state of the art of electrically controlled insulin delivery devices, applying electrical signals, highlighting advances, advantages, disadvantages, challenges, and innovations. Four different delivery strategies have been considered: (1) implantable devices, which are designed to attain sustained and long-term delivery; (2) transdermal insulin delivery; (3) injectable systems; and, finally, (4) combinations of groups 1 to 3.

Electrostimulable implantable devices are usually made of multilayered electrodes prepared by using layer-by-layer manufacturing techniques as film composites. Their success lies in the tunability of the characteristics of the components, the integration of insulin as a layer of bioactive agent, and innovative structural designs, which collectively enable personalized and localized treatment strategies. Although challenges persist, for example, manufacturing complexity and scalability, as well as the development of integrated electronics to provide insulin release triggered in response to a given biomarker (typically glucose), recent advances in productive engineering, nano/microfluidics, and electronics offer promising solutions.

The utilization of insulin-loaded polymeric microneedles for iontophoretic transdermal insulin delivery presents several advantages in comparison to passive transdermal administration, such as faster release of the drug into the skin and better control of the delivered dose. Furthermore, recent technological advances include the miniaturization of delivery systems as well as the incorporation of glucose detection to exploit the potential of closed-loop systems. Although the electric current applied in iontophoretic systems allows self-administration and does not cause cell damage, it also presents some risks, such as the possibility of skin irritation and burns due to a wrong choice of the electrodes.

On the other hand, the electroresponsive injectable systems that are beginning to be developed in the form of biohydrogels and NPs are highly promising. These systems allow not only for an immediate therapeutic response but also for precise control of insulin dosage over long periods of time. However, this kind of system is still in the early stages of development, and many questions remain unanswered; for instance, the effect of biodegradation products on the body, in the case of degradable hydrogels, or the elimination pathway followed by nonbiodegradable NPs.

The most emerging technology is the combination of several of the aforementioned strategies. Although this is undoubtedly a very promising approach, at least in theory, it is still too early to know whether it will be powerful enough to maximize the advantages and minimize the disadvantages of each of the individual strategies. In any case, recent advances in electrically stimulated insulin delivery systems highlight the interest in electrosensitive materials to regulate the release of this drug more quickly and with greater dose control, thus achieving more efficient diabetes therapy.
